# PET/MRI-guided GTV delineation during radiotherapy planning in patients with squamous cell carcinoma of the tongue

**DOI:** 10.1007/s00066-019-01480-3

**Published:** 2019-06-18

**Authors:** Natalia Samołyk-Kogaczewska, Ewa Sierko, Konrad Zuzda, Patryk Gugnacki, Piotr Szumowski, Małgorzata Mojsak, J. Burzyńska-Śliwowska, Marek Z. Wojtukiewicz, Kamil Szczecina, Dorota H. Jurgilewicz

**Affiliations:** 1 Department of Radiotherapy, Comprehensive Cancer Center, Bialystok, Poland; 2 Department of Oncology, Department of Radiotherapy, Medical University of Bialystok, Comprehensive Cancer Center, 12 Ogrodowa St., 15-027 Bialystok, Poland; 3 Scientific Student’s Association affiliated with Department of Oncology, Medical University of Bialystok, Bialystok, Poland; 4 Laboratory of Molecular Imaging–Nuclear Medicine Department, Medical University of Bialystok, Bialystok, Poland; 5 Department of Diagnostic Imaging, Comprehensive Cancer Center, Bialystok, Poland; 6 Department of Oncology, Medical University of Bialystok, Bialystok, Poland; 7 Department of Physics, Comprehensive Cancer Center, Bialystok, Poland

**Keywords:** PET/MRI, Radiation therapy, Gross tumor volume, Lingual carcinoma, Head and neck cancer, PET/MRT, Strahlentherapie, Makroskopisches Tumorvolumen, Zungenkarzinom, Kopf- und Halskarzinom

## Abstract

**Purpose:**

The aim of the study was to evaluate the usefulness and accuracy of 18-fluorine-labeled fluorodeoxyglucose (PET) and magnetic resonance imaging (MRI) hybrid in gross tumor volume (GTV) delineation during radiotherapy planning in patients with carcinoma of the tongue.

**Methods:**

Ten patients with squamous cell carcinoma (SCC) of the tongue underwent computed tomography (CT) and PET/MRI examination. The GTV for primary tumor and lymph nodes (nGTV) were defined on CT (GTV-CT) and compared to GTVs obtained from PET (GTV-PET) and MRI (GTV-MRI) images. Two methods of GTV determination were used: visual interpretation of CT, PET (GTV-PET_vis_) and MRI images and quantitative automatic method (Syngovia, Siemens) based on a chosen threshold value (20%, 30%, 40%, 50%) of standardized uptake values (SUV_max_) from PET examination (GTV-PET_20%_, GTV-PET_30%_, etc.). Statistical analysis of differences in GTV values obtained from CT, PET and MRI studies was performed. GTV-CT was used as a reference.

**Results:**

In all, 80% of GTV-MRI and 40% of GTV-PET_vis_ were larger than GTV-CT. Respectively, 20% of GTV-MRI and 60% of GTV-PET_vis_ were smaller than GTV-CT. Taking into account all threshold measurements, 70% of volumes were smaller than GTV-CT. GTV-PET_30%_ were the most closely related volumes to GTV-CT from all threshold methods in 50% of patients. GTV-PET_vis_ generated the most similar volumes in relation to GTV-CT from all PET measurements. Statistical analysis confirmed those results. Compared to nGTV-CT, 70% of nGTV-MRI and 20% of nGTV-PET_vis_ were larger. The remaining nGTV-MRI and nGTV-PET_vis_ measurements were smaller than nGTV-CT. Measurements of all thresholds nGTVs were smaller than nGTV-CTV in 52.5% of cases. nGTV-PET_20%_ were the most closely related volumes to nGTV-CT in 40% of the cases. Statistical analysis showed that nGTV-PET_20%_ (*p* = 0.0468), nGTV-PET_vis_ (*p* = 0.0166), and nGTV-PET_50%_ (*p* = 0.0166) diverge significantly from nGTV-CT results. nGTV-MRI (*p* = 0.1141), nGTV-PET_30%_ (*p* = 0.2845), and nGTV-PET_40%_ (*p* = 0.5076) were significantly related with nGTV-CT.

**Conclusion:**

Combination of PET/MRI provides more information during target tumor mass delineation in radiotherapy planning of patients with SCC of the tongue than other standard imaging methods. The most frequently matching threshold value was 30% of SUV_max_ for primary tumor delineation and 30–40% of SUV_max_ for nGTV determination.

**Electronic supplementary material:**

The online version of this article (10.1007/s00066-019-01480-3) contains supplementary material, which is available to authorized users.

## Zusammenfassung

**Zielsetzung:**

Die Studie hatte zum Ziel, die Nützlichkeit und Genauigkeit der Kombination aus ^18^F-Fluordesoxyglukose-Positronenemissionstomographie (PET) und Magnetresonanztomographie (MRT) bei der Bestimmung des makroskopischen Tumorvolumens (GTV) während der Bestrahlungsplanung bei Patienten mit Zungenkarzinom zu bewerten.

**Methoden:**

Eine Gruppe von 10 Patienten mit Plattenepithelkarzinom (SCC) der Zunge wurde einer Computertomographie (CT) und einer PET/MRT-Untersuchung unterzogen. Die GTV für Primärtumor und Lymphknoten (nGTV) wurden mithilfe der CT (GTV-CT) definiert und mit GTV aus PET- (GTV-PET) und MRT-Bildern (GTV-MRT) verglichen. Es wurden zwei Methoden zur GTV-Bestimmung verwendet: die visuelle Interpretation von CT-, PET- (GTV-PET_vis_) und MRT-Bildern sowie die quantitative automatische Methode (syngo.via, Siemens) auf Basis eines ausgewählten Schwellenwerts (20%, 30%, 40%, 50%) von maximalen „standardized uptake values“ (SUV_max_) aus der PET-Untersuchung (GTV-PET_20%_, GTV-PET_30%_ usw.). Es wurde eine statistische Analyse der Unterschiede in den GTV-Werten aus CT-, PET- und MRT-Untersuchungen durchgeführt. Die GTV-CT wurde als Referenz verwendet.

**Ergebnisse:**

Insgesamt 80% der GTV-MRT und 40% der GTV-PET_vis_ waren größer als GTV-CT. Entsprechend waren 20% der GTV-MRT und 60% der GTV-PET_vis_ kleiner als GTV-CT. Unter Berücksichtigung aller Schwellenwerte waren 70% der Volumina kleiner als GTV-CT. Die GTV-PET_30%_ standen unter allen Schwellenwertverfahren bei 50% der Patienten am engsten mit GTV-CT in Beziehung. GTV-PET_vis_ erzielten aus allen PET-Messungen die ähnlichsten Volumina in Bezug auf GTV-CT. Die Ergebnisse wurden durch die statistische Analyse bestätigt. Im Vergleich zu nGTV-CT waren 70% der nGTV-MRT und 20% der nGTV-PET_vis_ größer. Die übrigen nGTV-MRT- und nGTV-PET_vis_-Messungen fielen kleiner aus als die nGTV-CT. Die Messungen aller Schwellenwert-nGTV waren in 52,5% der Fälle kleiner als nGTV-CTV. nGTV-PET_20%_ standen in 40% der Fälle am engsten mit nGTV-CT in Beziehung. Die statistische Analyse zeigte, dass nGTV-PET_20%_ (*p* = 0,0468), nGTV-PET_vis_ (*p* = 0,0166) und nGTV-PET_50%_ (*p* = 0,0166) signifikant von den nGTV-CT-Ergebnissen abweichen. nGTV-MRT (*p* = 0,1141), nGTV-PET_30%_ (*p* = 0,2845) und nGTV-PET_40%_ (*p* = 0,5076) waren am signifikantesten mit nGTV-CT verbunden.

**Schlussfolgerung:**

Die Kombination von PET und MRT bietet eine bessere Genauigkeit bei der Zieltumorabgrenzung im Rahmen der Bestrahlungsplanung bei Patienten mit SCC der Zunge als andere Standardverfahren der Bildgebung. Der am häufigsten übereinstimmende Schwellenwert war 30% von SUV_max_ für die primäre Tumorabgrenzung und 30–40% von SUV_max_ für die nGTV-Bestimmung.

## Introduction

Head and neck cancers (HNC) are the sixth most common malignancy in the world [1]. Squamous cell carcinoma (SCC) is the major histology. Radiotherapy alone or with concurrent chemotherapy/immunotherapy, beside surgery, is the main treatment method of HNC patients [2]. During the last 20 years impressive technological progress in radiation oncology has been observed. Techniques characterized by high-precision dose delivery to the tumor, with maximal sparing of normal tissues, have been introduced to every-day practice including, among others, intensity modulated radiotherapy (IMRT), volumetric modulated arc therapy (VMAT) and stereotactic radiotherapy/radiosurgery (SRT/SRS). Accurate definition of the target volumes is crucial in these techniques. Spatial error in volume delineation may result in early recurrence due to undertreatment near the tumor boundary or unnecessary damage to critical anatomical structures [2–4].

In clinical practice, target volumes are based on information obtained from different imaging methods. Gross tumor volume (GTV) is defined as the visually determined tumor directly on images of a chosen study. Accuracy in target volume delineation depends, inter alia, on the imaging method [3]. Computed tomography (CT) and magnetic resonance imaging (MRI) are the most frequently chosen anatomical imaging modalities. Currently, the use of molecular imaging, mainly positron emission tomography with 18-fluorine-labeled fluorodeoxyglucose (^18^F-FDG PET), continues to grow [5]. ^18^F-FDG PET allows visualization of foci with increased glucose uptake, which is, among others, characteristic for carcinomas [6]. Images obtained from ^18^F-FDG PET have high contrast, but low spatial resolution. Because of this, ^18^F-FDG PET is combined with morphological imaging methods, like CT or MRI [3]. Magnetic resonance imaging has a potential advantage over CT, since it is characterized by excellent soft tissue contrast, which allows for detection of infiltration of adjacent structures or perineural and vascular spread [7]. The prospective study by Lonneux et al. [8] showed that the combination of ^18^F-FDG PET/CT compared to CT alone is more accurate and has higher sensitivity, especially in evaluation of metastatic lymph nodes. The advantage of PET/CT and MRI over CT alone encouraged the investigation of the value of images obtained from PET/MRI hybrid in target volume delineation. To date, a limited number of studies have assessed the use of this innovative hybrid combination of molecular and anatomical imaging in radiotherapy planning [7].

The two most frequently used methods of GTV metabolic determination include visual interpretation and an automatic method based on an established threshold of standardized uptake value (SUV), defined as the percentage of maximal SUV (% SUV_max_) [9]. The higher threshold value limits the tumor volume to tissues with more extensive metabolism and cell proliferation [10].

The aim of the study was to evaluate the usefulness and accuracy of PET/MRI hybrid in GTV delineation during radiotherapy planning in patients with carcinoma of the tongue.

## Materials and methods

A retrospective analysis of anatomical (CT and MRI) and metabolic (PET) studies was performed on a group of 10 patients with histologically proven squamous cell carcinoma (SCC) of the tongue. Other inclusion criteria were age over 18 years old, glycemic blood level under 160 mg/dl, lack of uncontrolled systemic diseases, lack of hypersensitivity or allergic reaction on intravenous contrast or ^18^F-FDG in the past, or lack of presence of metal elements in the patient’s body (cardiac pacemakers, cochlear implants, intrauterine contraceptive devices, metal shavings in an eyeball, surgical clips, metal surgical stitches).

Patients had a median age of 55 years (range 36–66 years; 5 women, 5 men). Clinical stage of the disease ranged form I to IVa, based on physical examination and CT images. Patient characteristics are presented in Table 1. No distant metastases were revealed.

**Table 1:** Characterization of lingual squamous cell carcinoma (SCC) patients according to histopathology (H-P), histology grade score, clinical stage (TNM classification, AJCC, ed. 8, 2017), based on computed tomography (CT) evaluation, status of human papilloma virus (HPV) infection, smoking and biopsy performed before positron emission tomography/magnetic resonance (PET/MRI) imaging

No. of pts	H-P	Histology grade score	TNM	HPV status (+/−)	p16 status (+/−)	EBV status (+/−)	Ki67 (%)	Smoking status	Biopsy before PET/MRI (days)
1	SCC	2	T4N3M0	−	−	−	30	Yes	No
2	SCC	2	T2N0M0	−	−	−	30	Yes	No
3	SCC	2	T3N2cM0	N/A	N/A	N/A	N/A	Yes	Yes (25)
4	SCC	2	T3N2cM0	N/A	N/A	N/A	N/A	No	No
5	SCC	2	T3N0M0	N/A	N/A	N/A	N/A	Yes	No
6	SCC	2	T2N1M0	−	−	−	20	No	Yes (17)
7	SCC	2	T4N1M0	−	−	−	30	No	Yes (15)
8	SCC	2	T2N1M0	−	−	−	30	Yes	No
9	SCC	1	T4N2bM0	+	+	−	30	Yes	Yes (20)
10	SCC	2	T1N0M0	−	−	+	50	No	Yes (14)

*N/A* not available, *pts* patients

The group of patients was homogeneous in terms of biochemical parameters (e.g., glycemia level, blood morphology, C‑reactive protein (CRP) concentration, thyroid hormones level, renal and liver function parameters, electrolyte blood concentration).

Every patient routinely underwent CT examination of the head and neck region, with intravenous contrast (Ultravist 300, 1 ml/kg) on 320-slices CT scanner (Aquilion ONE, Canon Medical Systems Corporation, Otawara, Japan).

After an average of 8 days (range 7–13 days) PET/MRI studies were performed on a 3 T Siemens Biograph mMRI scanner (Siemens Healthcare GmbH, Erlangen, Germany). ^18^F-FDG (4 MBq/kg, range 242–404 MBq ^18^F-FDG per patient) was administered intravenously and the average time between tracer injection and the start of PET scanning was 60 min. The PET/MRI examination consisted of a low-resolution, nondiagnostic whole-body MRI scan, followed by PET scanning and diagnostic MRI scanning (T1- and T2-weighted sequences and contrast enhancement sequences) of the head and neck region (integrated parallel acquisition technique factor 2, acquisition time 19 s, 3.12 mm slice thickness, 20% interslice gap, 192 × 121 matrix, 500 mm × 328 mm field of view [FOV], repetition time 3.6 ms, echo time 1.23 and 2.46 ms).

GTVs were defined on PET images (GTV-PET) and compared to the GTVs obtained from CT (GTV-CT) and MRI (GTV-MRI). Two methods of GTV definition were used in this study: visual interpretation of CT, PET, and MRI images as well as an automatic method based on a chosen threshold value of SUV_max_ from PET examination.

Volumetric evaluations were performed using Siemens *syngo.via* VB10B software (Siemens Healthcare GmbH, Erlangen, Germany) on a HP Z420 working station (Hewlett-Packard Development Company, L.P., Houston, TX, USA). Visual interpretation was made in cooperation with a radiologist, a nuclear medicine specialist and a radiation oncologist. GTV delineation was created using a Wacom Intuos Draw graphics tablet (Wacom Co Ltd, Saitama, Japan). The primary tumor and lymph nodes (>10 mm in shortest dimension) were delineated on contrast enhancement CT scans and T1-weighted VIBE (volumetric interpolated breath-hold examination) Dixon MRI sequence. Manually created target volumes on PET images (GTV-PET_vis_) were obtained using “halo” method based on *spectrum* window level in *syngo.via* software. “Halo” [11] was recognizable by a specific color, slim wall, low SUV area located around the region of the maximal metabolic activity of the tumor/lymph node.

**Fig. 1:**
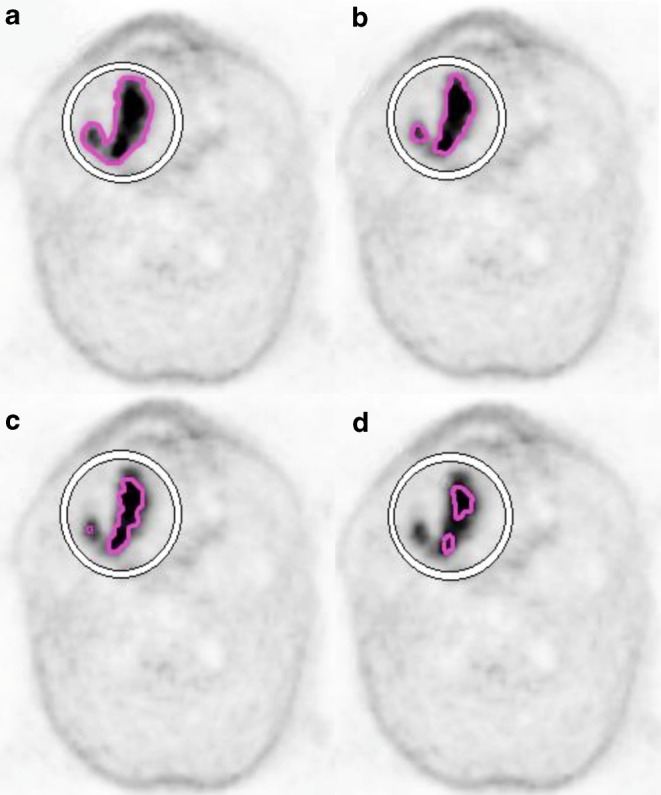
Comparison of primary tumor (GTV, gross tumor volume) delineation using automatic fixed threshold method in patient with squamous cell carcinoma of the tongue (T3N2bM0): **a** GTV obtained with threshold of 20% of SUV_max_ (maximum standardized uptake value), **b** threshold of 30% of SUV_max_, **c** threshold of 40% of SUV_max_, **d** threshold of 50% of SUV_max_

The fixed thresholds method was based on an automatic contour function—volume of interest (VOI) isocontour. A “sphere” was placed over the high uptake region and the contour was obtained by applying selected SUV_max_ thresholds of 20%, 30%, 40%, 50%, and 60% and obtained volumes were named GTV-PET_20%_, GTV-PET_30%_, GTV-PET_40%_, GTV-PET_50%_ and GTV-PET_60%_, respectively (Fig. 1). Volumes of 60% threshold were inappropriately small in comparison to GTV-CT, so they were excluded from further evaluation.

Obtained volumes of GTV from CT, MRI and PET were compared. Statistical analyses were performed. The GTV-CT was used as a reference because CT is fundamental in the majority of currently used radiotherapy planning systems. Moreover, most guidelines for GTV and organs at risk delineation in head and neck region published by the most influential radiation oncologist associations are based on evaluation of CT images [12, 13]. The average and median were calculated for obtained results. The level of significance was considered as *p* < 0.05. The evaluation of normality of distribution was carried out with the Shapiro–Wilk test. The data were compared with the Wilcoxon pair test.

Moreover, spatial analysis between GTV-MRI, GTV-PET_vis_ and GTV-CT was performed. Images from CT, MRI and PET study were fused using mutual information as registration method in Oncentra (Nucletron, Veenendaal, Netherlands). The Dice similarity coefficient (DSC) and the modified Hausdorff distance (mHD) were calculated. The DSC was calculated using the equation: 2 × (A∩B) / (A + B), where A and B represent two volumes, (A∩B) represents the volume of intersection, and (A + B) represents the absolute sum of their volumes [7]. The A DSC values are between 0 and 1, where a DCS of 0 indicates no spatial overlap at all and a DSC of 1 indicates complete overlap. The mHD [14] measures the similarity between two volumes by reporting the mean orthogonal distance between surface points.

Registration accuracy was qualitatively measured by the DSC and mHD computed on segmented tumors on CT and hybrid PET/MRI (GTV-PET/MRI) images in *syngo.via* (Siemens Healthcare GmbH, Erlangen, Germany).

The study was performed in accordance with the ethical standards laid down in the 1964 Declaration of Helsinki and its later amendments and was approved by the Bioethical Committee of the Medical University of Białystok, Poland.

## Results

### Tumor GTV

Results of the volumetric assessment of primary tumor volumes (GTV) obtained from CT, MRI and PET images are shown in Table 2. Measurements of primary tumor SUV_max_, SUV_mean_ and correction of SUV values for healthy soft tissue are presented in Table 3.

**Table 2:** Results of the volumetric assessments of primary tumor volumes (gross tumor volume, GTV) obtained from computed tomography (CT), magnetic resonance (MRI) and 18-fluorine-labeled fluorodeoxyglucose positron emission tomography (18F-FDG-PET) images in each patient (1–10)

Pts	GTV CT cm^3^	GTV MRI cm^3^ (%)	GTV PET _vis_ cm^3^ (%)	GTV PET_20%_ cm^3^ (%)	GTV PET_30%_ cm^3^ (%)	GTV PET_40%_ cm^3^ (%)	GTV PET_50%_ cm^3^ (%)
1	26.4	26.84 (101.7)	*18.56* *(70.3)*	**17.31** (65.6)	11.79 (44.6)	8.63 (32.7)	6.35 (24)
2	32.12	60 (186.8)	59.32 (184.7)	103.53 (322.3)	66.9 (208.3)	48.45 (150.8)	***36.72*** *(114.3)*
3	12.16	11.4 (93.7)	*12.32* *(101.3)*	27.74 (228.1)	**9.33** (76.7)	4.65 (38.2)	2.84 (23.3)
4	82.23	93.59 (113.8)	100.6 (122.3)	***94.07*** *(114.4)*	60.42 (73.5)	47.09 (57.3)	35.59 (43.3)
5	20.23	23.88 (118)	*15.02* *(74.2)*	**14.49** (71.6)	9.26 (45.77)	6.59 (32.6)	4.63 (22.9)
6	5.66	6.53 (115.4)	*5.91* *(104.4)*	14.08 (248.8)	**5.18** (91.5)	3.58 (63.2)	2.67 (47.2)
7	4.08	4.54 (111.3)	1.73 (42.4)	22.95 (562.5)	9.13 (223.8)	***2.51*** *(61.5)*	1.40 (34.3)
8	19.51	13.19 (67.6)	11.82 (60.6)	33.57 (172.1)	***13.16*** *(67.4)*	5,59 (28.6)	2.68 (13.7)
9	4.35	6.23 (143.2)	3.26 (74.9)	7.77 (178.6)	***3.48*** *(80)*	2.24 (51.5)	1.42 (32.6)
10	4.78	6.54 (136.8)	*3.39* *(70.9)*	7.17 (150)	**2.59** (54.2)	1.48 (31)	0.95 (19.9)

*Italised* values of PET-based volumes the most closely related to CT-based volumes, *bold* *+bold italic* values of PET-based volumes obtained from fixed threshold method, which are the most closely related to CT-based volumes, *pts* patients

*%* percentage of CT-based volume, *PET*_*vis*_ visual method, *PET*_*20%*_*, PET*_*30%*_*, PET*_*40%*_*, PET*_*50%*_ volumes covered by 20%, 30%, 40%, 50% threshold of SUV_max_, respectively

**Table 3:** Results of the assessments of maximal and mean standardized uptake value (SUV) of primary tumor and mean SUV of soft tissue obtained from 18-fluorine-labeled fluorodeoxyglucose positron emission tomography (^18^F-FDG-PET) images in patients (1–10) with squamous cell carcinoma of the tongue

No. of pts	Tumor SUV_max_	Tumor SUV_mean_	Soft tissue SUV_mean_	Tumor SUV_max_/soft tissue index	Tumor SUV_mean_/soft tissue index
1	12.3	7.26	0.44	27.95	16.5
2	8.05	5.1	0.5	16.1	10.2
3	9.71	5.63	0.6	16.18	9.38
4	13.7	8.73	0.52	26.35	16.79
5	20.6	12.4	0.67	30.75	18.51
6	8.47	5.43	0.39	21.72	13.92
7	4.66	2.61	0.49	9.51	5.33
8	9.26	5.09	0.54	17.15	9.43
9	12.1	7.09	0.63	19.21	11.25
10	11.4	6.7	0.5	22.8	13.4

*SUV*_*max*_ maximal standardized uptake value, *SUV*_*mean*_ mean standardized uptake value, *pts *patients

Most of GTV-MRI (80%) and 40% of GTV-PET_vis_ were larger than the reference GTV-CT. Respectively, 20% of tumor volumes obtained from MRI and 60% of GTV-PET_vis_ were smaller than GTV-CT.

**Fig. 2:**
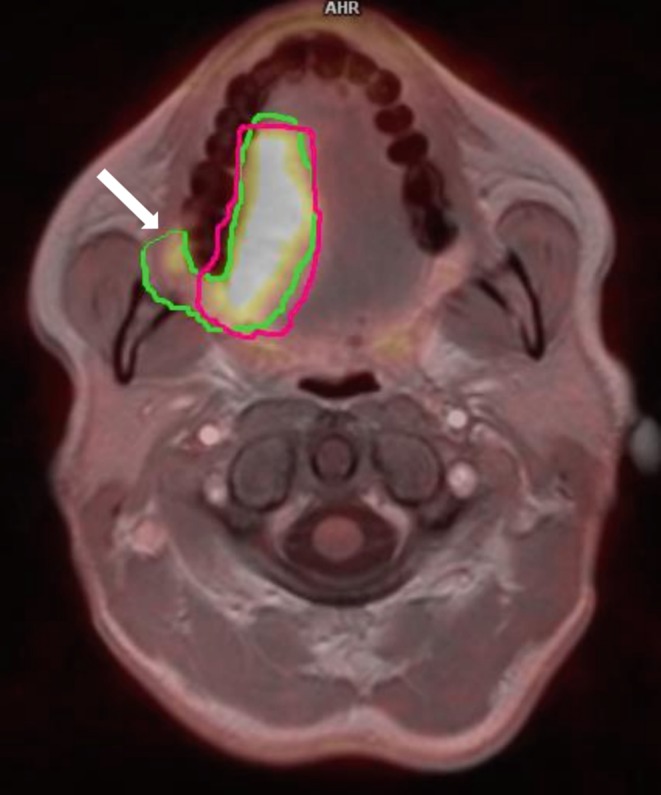
Primary tumor volume (GTV, gross tumor volume) delineated with manual method and presented on fusion of 18-fluorine-labeled fluorodeoxyglucose positron emission tomography (PET) and magnetic resonance (MRI) images. PET-based GTV (*green line*) are larger than MRI-based GTV (*pink line*) and include tumor’s infiltration on the retromandibular triangle (*arrow*)

In 8 out of 10 patients GTV-MRI contained a smaller GTV-PET_vis_. In two cases some mismatches were observed. In one patient GTV-PET_vis_ was distinctly (about 7%) larger than GTV-MRI and PET showed tumor infiltration on adjacent structures of oropharynx, invisible in the MR study. In another case, GTV-PET_vis_ and GTV-MRI were similar, but on PET images the retromandibular triangle’s infiltration was much better visualized (Fig. 2).

Taking into account all threshold measurements, 70% of volumes were smaller than referenced GTV-CT (Table 2). The most closely related GTV values—from all GTV based on the threshold method—were generated from 30% SUV_max_ threshold and this was the case for 50% of patients. Analyzing all values obtained from PET measurements (GTV-PET_vis_; GTV-PET_20%_; GTV-PET_30%_; GTV-PET_40%_; GTV-PET_50%_), the visual method generated the most similar volumes in relation to GTV-CT.

**Fig. 3:**
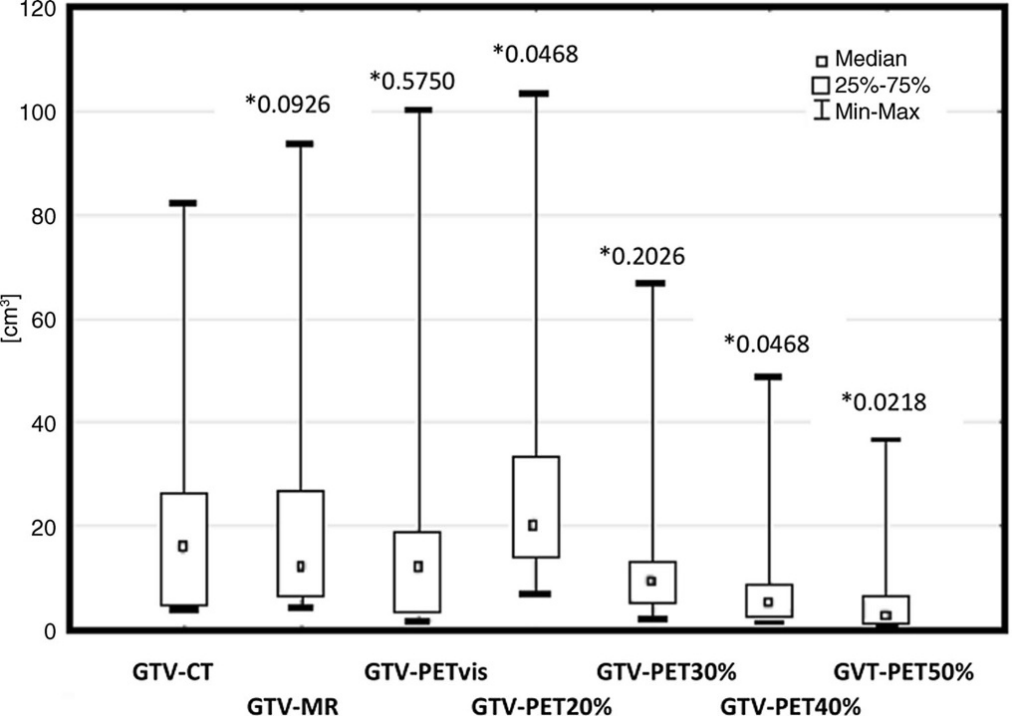
Statistical comparison of primary tumor volumes (gross tumor volume, GTV) delineated using visual method and fixed threshold method, obtained from computed tomography (CT), magnetic resonance (MRI) and 18-fluorine-labeled fluorodeoxyglucose positron emission tomography (PET) images. The graph shows the median, the average and the level of statistical significance *p* (*asterisk*) of obtained results. *Min* minimal value, *Max* maximal value, *vis* visual method of GTV delineation, *PET*_*20%*_*, PET*_*30%*_*, PET*_*40%*_*, PET*_*50%*_ volumes covered by 20%, 30%, 40%, 50% threshold of SUV_max_, respectively

The average, the median and the standard deviation of tumor’s GTV are presented in Fig. 3.

Statistical analysis confirmed earlier observations. Similar results were obtained in GTV-MRI (*p* = 0.1141), GTV-PET_30%_ (*p* = 0.2026) and GTV-PET_vis_ (*p* = 0.5750). On the other hand, GTV-PET_20%_ (*p* = 0.0468), GTV-PET_40%_ (*p* = 0.0468) and GTV-PET_50%_ (*p* = 0.0218) diverge statistically significantly from referenced GTV-CT results (Fig. 1).

The average value of DSC for GTV-CT and GTV-MRI was 0.74 (range 0.66–0.85) and for GTV-CT and GTV-PET_vis_—0.72 (range 0.57–0.79). Average mHD between GTV-CT and GTV-MRI was 13.2 mm (range 4–19 mm) and between GTV-CT and GTV-PET_vis_—12.4 mm (range 5–21 mm). The registration accuracy measurements in the form of mHD and DSC values for GTV-CT and GTV PET/MRI was in the range of 0–28 mm (average 16.2 mm) and 0–0.82 (average 0.55), respectively. The DSC and mHD for GTV-MR, GTV-PET_vis_, GTV-PET/MRI and GTV-CT are presented in Table 4.

**Table 4:** Volume agreement between gross tumor volume (GTV) obtained from computed tomography (GTV-CT), magnetic resonance (GTV-MRI), positron emission tomography (GTV-PET_vis_) and automatic fused positron emission tomography/magnetic resonance (GTV-PET/MRI) in patients with squamous cell carcinoma of the tongue (1–10)

No. of pts	mHD [mm] between:			DCS for:		
	GTV-CT and GTV-MRI	GTV-CT and GTV-PET_vis_	GTV-CT and GTV PET/MRI	GTV-CT and GTV-MRI	GTV-CT and GTV-PET_vis_	GTV-CT and GTV PET/MRI
1	14	15	21	0.74	0.70	0.5
2	19	19	20.3	0.68	0.78	0.64
3	18	21	20.6	0.71	0.79	0.82
4	17	20	25	0.8	0.79	0.6
5	18	8	28	0.85	0.73	0.4
6	10	8	10.4	0.73	0.68	0.75
7	12	8	12.2	0.66	0.73	0.67
8	12	12	20	0.67	0.57	0.3
9	4	5	4.1	0.77	0.72	0.77
10	8	8	0	0.75	0.66	0
**Average**	**13.2**	**12.4**	**16.2**	**0.74**	**0.72**	**0.55**

*pts* patients, *PET*_*vis*_ visual method of GTV delineation, *DSC* Dice similarity coefficient, *mHD* modified Hausdorff distance

### Nodal GTV

In the study, only enlarged, exceeding 10 mm in the shortest transverse dimension or round-shaped lymph nodes with contrast enhancement on CT or MRI and/or increased ^18^FDG uptake in PET were taken into account. The number of detected lymph nodes in each imaging study differed. On CT images—22 lymph nodes were detected, on MRI—20 and on PET—15. Only 10 metastatic lymph nodes were suspicious for malignancy in all examined imaging techniques and this series was taken into account in further analysis.

Results of the volumetric assessments of lymph node target volumes (nGTV) obtained from CT (nGTV-CT), MRI (nGTV-MRI) and PET (visual method: nGTV-PET_vis_ and fixed threshold values: nGTV-PET_20%_; nGTV-PET_30%_; nGTV-PET_40%_; nGTV-PET_50%_) are shown in Table 5.

**Table 5:** Results of the volumetric assessments of lymph nodes volumes (nodal gross tumor volume, nGTV) obtained from computed tomography (CT), magnetic resonance (MRI) and 18-fluorine-labeled fluorodeoxyglucose positron emission tomography (18F-FDG–PET) images in each patient (1–10)

No. of pt	nGTV CT cm^3^	nGTV MRI cm^3^ (%)	nGTV PET _vis vis_ cm^3^ (%)	nGTV PET_20%_ cm^3^ (%)	nGTV PET_30%_ cm^3^ (%)	nGTV PET_40%_ cm^3^ (%)	nGTV PET_50%_ cm^3^ (%)
1	*4*	*9.14 (228.5)*	*5.07 (126.7)*	***3.62*** * (90.5)*	*2.36 (59)*	*1.61 (40.3)*	*1.02 (22.5)*
	1.56	3.54	0	–	–	–	–
	2.11	4.31	0	–	–	–	–
	1.59	0	0	–	–	–	–
2	0	4.18	2.35	–	–	–	–
	*4.19*	*4.13 (98.6)*	*1.34 (32)*	*10.37 (247.5)*	*8.68 (207.2)*	*6.15 (146.8)*	***3.56*** * (85)*
	*1.52*	*2.42 (159.2)*	*0.39 (59.3)*	*8.54 (561.8)*	*5.54 (364.5)*	*3.16 (207.9)*	***2.02*** * (132.9)*
	1.43	0	0	–	–	–	–
3	*5.8*	*2.11 (36.4)*	*0.77 (13.3)*	***3.94*** * (68)*	*2.14 (36.9)*	*0.99 (17.1)*	*0.44 (7.6)*
	2.27	2.55	0	–	–	–	–
	0.78	1.46	0	–	–	–	–
	0	0	0.47	–	–	–	–
4	*5.3*	*8.09 (152.6)*	*4.9 (92.5)*	*23.13 (436.4)*	*9.26 (174.7)*	***5.54*** * (104.5)*	*3.26 (61.5)*
	*1.8*	*3.01 (162.2)*	***1.6*** * (88.8)*	*9.66 (536.7)*	*7.76 (431.1)*	*4.55 (252.8)*	*2.23 (123.9)*
	0.65	0	1.5	–	–	–	–
	0.67	0	0	–	–	–	–
	0.4	0	0	–	–	–	–
5	*5.6*	*6.2 (110.7)*	*2.5 (44.6)*	***5.14 *** *(91.8)*	*2.31 (41.25)*	*1.27 (22.7)*	*0.69 (12.3)*
	*1.9*	*3.46 (182.1)*	*0.6 (31.6)*	*4.41 (232.1)*	*3.13 (164.7)*	***1.78 *** *(93.7* ***)***	*1.01 (53.2)*
	0	1.48	0.8	–	–	–	–
	0	1.2	0	–	–	–	–
	1.38	0	0	–	–	–	–
6	0	0	0	–	–	–	–
7	0	0	0	–	–	–	–
8	*1.06*	*2.14 (201.9)*	*0.88 (83)*	*2.57 (242.5)*	*1.76 (166)*	***1.03 *** *(97.2* ***)***	*0.59 (55.7)*
	*2.58*	*2.06 (79.8)*	*0.69 (26.7)*	***3.49*** * (135.3)*	***1.48 *** *(57.4)*	*0.73 (28.3)*	*0.38 (14.7)*
9	0.86	1.14	0	–	–	–	–
	1.2	1.67	0	–	–	–	–
10	0	1.98	0.69	–	–	–	–

*italicized* volumes of lymph nodes detected with every of three imaging methods: CT, MRI and PET, *italic bold* values of PET-based volumes obtained from fixed threshold method, which are the most closely related to CT-based volumes

*%* percentage of CT-based volume, *PET vis* visual method, *PET20%/PET30%/PET40%/PET50%* fixed threshold method, *pts* patients

Comparing to standard nGTV-CT—70% of nGTV-MRI and 20% of nGTV-PET_vis_ were larger. Remaining nGTV-MRI and nGTV-PET_vis_ measurements were smaller than nGTV-CT.

Measurements of all thresholds nGTVs were smaller than nGTV-CTV in 52.5% of cases. nGTV-PET_20%_ were the most closely related volumes to nGTV-CT target volumes and this has been observed in 40% of the cases.

**Fig. 4:**
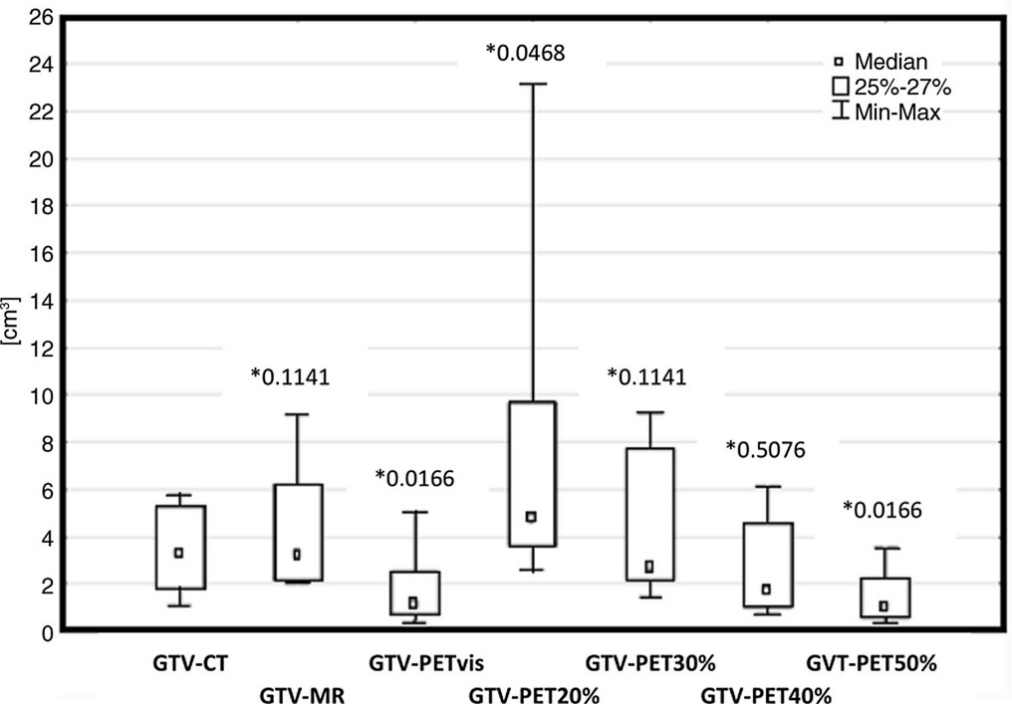
Statistical comparison of lymph nodes volumes (nodal gross tumor volume, nGTV) delineated using visual method and fixed threshold method, obtained from computed tomography (CT), magnetic resonance (MRI) and 18-fluorine-labeled fluorodeoxyglucose positron emission tomography (PET) images. The graph shows the median, the average and the level of statistical significance *p* (*asterisk*) of obtained results. *Min* minimal value, *Max* maximal value, *vis* visual method of GTV delineation, *PET*_*20%*_*, PET*_*30%*_*, PET*_*40%*_*, PET*_*50%*_ volumes covered by 20%, 30%, 40%, 50% threshold of SUV_max_, respectively

On the other hand, statistical analysis (Fig. 2) showed that nGTV-PET_20%_ (*p* = 0.0468) diverge significantly from nGTV-CT results, as well as nGTV-PET_vis_ (*p* = 0.0166) and nGTV-PET_50%_ (*p* = 0.0166). nGTV-MRI (*p* = 0.1141), nGTV-PET_30%_ (*p* = 0.2845) and nGTV-PET_40%_ (*p* = 0.5076) were the most significantly related with nGTV-CT. The average, the median and the standard deviation of nodal GTV are also presented in Fig. 4.

## Discussion

^18^FDG-PET has been increasingly used in radiation oncology since integration of ^18^FDG-PET and CT imaging, which allows for simultaneous utilization of metabolic and anatomic data. Furthermore, in patients with lung cancer, ^18^FDG-PET has an established role in target delineation [15]. Contrary, in HNC there is no consensus about the role of ^18^FDG-PET for GTV determination so far. Moreover, there are a limited number of publications regarding the use of modern hybrid ^18^FDG-PET/MRI in target delineation in HNC patients. Magnetic resonance imaging is characterized by excellent soft tissue contrast [7, 9]. PET data might identify a tumor region not clearly visible on other imaging studies and prevent geographical misstatements, especially in oral cavity cancers [16]. This imaging method provides useful information about the biologic behavior of tumors and, when incorporated into radiation treatment planning, may allow for personalized radiation plans for each patient. It can be used to generate tailored “dose painting” that allows for different doses to be delivered to separate subvolumes of the tumor [17]. Combining the advantages of both ^18^FDG-PET and MRI may provide better accuracy of GTV delineation than other imaging techniques [9].

Multiple methods were proposed for accurate contouring of GTV in ^18^FDG-PET-guided radiotherapy [2]. The two most popular methods—visual and automatic fixed threshold method—were chosen in the study.

In terms of technological aspects, the visual contouring method is less demanding. It does not require any specialist equipment, except a standard treatment planning station. On the other hand, this method is highly observer-dependent [2, 9]. During contouring on PET images, settings of different window levels would result in significantly different target volumes [2]. One of the techniques of manual delineation on PET scans is the “halo” method. The use of the “halo” by Ashamalla et al.[11] resulted in reduction of interobserver variability and in modification of GTV-PET in 53% of cases compared with GTV-CT. Visual planning relies on the physician’s experience in recognition of various processes that led to physiological uptake of ^18^FDG in the head and neck region [18, 19]. In addition, the anatomical boundaries and location, clinical situation, patient motion during examination and other artifacts have great influence on the quality of contouring [20, 21].

Results obtained from manual delineation showed that the primary tumor volumes from MRI, PET and CT differ slightly from each other. Differences were statistically irrelevant. However, in particular patients these differences were significant. In 80% of patients, GTV-MRI were larger than in the reference GTV-CT. It might be a result of better soft tissue imaging and more accurate definition of the tumor’s infiltration boundary. Similar results were obtained by Ahmed et al. [22], who used T1-weighted post-contrast MRI sequences to delineate GTVs in cases of tumors of the base of the tongue. Another cause of these differences can be found in program’s sequence (*mMR General*), in which PET/MRI and CT images were viewed and which is dedicated mainly for working with PET/MRI images. Delouya et al. [15] reported that in most cases GTV of the primary tumor was smaller when delineated on ^18^FDG PET/CT vs. CT alone. The same results were described by other authors [23, 24]. In our study, 60% from GTV-PET_vis_ are smaller than GTV-CT. Bruella et al. [2] demonstrated that the difference between GTV obtained from PET and from CT was not statistically significant. Other investigators [7] revealed that PET/MRI-based and CT-based target volumes were similar in cases of primary oropharyngeal tumors. On the other hand, Ma et al. [9] reported that GTV-PET_vis_ were larger than GTV-MRI when delineating on hybrid PET/MRI images. According to the authors, it was related to delineation of GTV-MRI on three-dimensional T2-weighed TSE (turbo spin echo) images and with the application of the “halo” method. In our study, 40% of primary tumor volumes based on PET were larger compared to volumes from CT alone. This results may be related to many artifacts from dental fillings present in CT studies. Artifacts veiled areas of the tongue and the floor of the mouth which significantly impeded target delineation. PET/MRI was performed on average 8 days after CT, so patient’s position during each examination could be slightly different. Moreover, imaging studies were performed without immobilization of the head and this also can be a reason of spatial discrepancies in GTV delineation.

The fixed threshold method is one of the most commonly used methods of target volume determination. The advantage is in reduction of time required for manual delineation. This method does not depend on window level. The use of a threshold of 40% and 50% of SUV_max_ is predominantly reported in the literature [7, 9, 16, 18, 21, 25–33]. In one of the first studies assessing the use of ^18^FDG-PET in GTV delineation, Ciernik et al. [10] pointed that the best threshold value for the clinical setting is 50% of SUV_max_. Other authors [16] reported that volumes obtained from thresholds within the range of 20–41% of SUV_max_ were comparable to GTV-CT. The results of our study do not allow the identification of the only one threshold value for target delineation either. None of the tested threshold values (20%, 30%, 40%, 50%) determined volumes similar to the reference GTV-CT in all cases. Threshold volumes of 30% SUV_max_ are most comparable in the largest number (50%) of patients, which was confirmed in a statistical analysis. Ma et al. [9] reported that 31 ± 11.17% is the best threshold value for determination of primary tumor volumes. In our study, 70% of obtained volumes based on the fixed threshold method are smaller than GTV-CT. This is comparable with Paulinio et al. [25], who documented that 75% of GTV-PET_30%_ were smaller than GTV-CT. Other authors obtained similar results: all threshold-based GTV-PET were smaller than GTV-CT [32]. The lack of one, fixed threshold value may lay in the absence of uniformity of ^18^FDG uptake, which can be related to the presence of areas of hypoxia or necrosis, especially in advanced tumors [26]. Some authors argue that accuracy of the automatic threshold method is highly dependent on tumor characteristics [27]. For example, a 40% threshold of SUV_max_ generates inappropriately large volumes with low avidity tumors and small volumes with high avidity tumors. In the research of Guardia et al. [28], GTV-PET obtained from the threshold method were slightly larger than volumes from conventional imaging. They explained it as being due to anatomic imprecision. PET images often falsely include air in the GTV or are distorted due to patient’s involuntary breathing or swallowing, because acquisition of these images takes several minutes.

Beside the contouring method, in our study ^18^FDG-PET helped to detect tumor infiltration on structures, which were not visible on CT and hardly visible on MRI. Bruela et al. [2] came to a similar conclusion, namely that PET has the potential to identify tumor volume outside GTV-CT. In another study, the matching rate was good—about 90% volumes of GTV-PET was overlapped to GTV-MRI but 10% of tumor and lymph node volumes obtained from PET was outside GTV-MRI [9]. For spatial analyses of GTV obtained from different imaging method we used DSC and mHD. DSC has a limited range (0–1), where 0 indicates no spatial overlap and DSC of 1 indicates perfect overlap. The higher the DICE index (i.e. >0.5) is, the higher the agreement [21]. Some investigators reported DSC 0.7 as a “good” overlap, noting that DSC may vary more with changes in the size and less with the shape of the compared volumes [34]. Our results show that spatial compliance between GTV-CT/GTV-MRI and GTV-CT/GTV-PET_vis_ is rather high, because mean DSC values for both compared GTVs pairs were above 0.7 (0.74 and 0.72, respectively). On the other hand, mHD is best for matching two objects based on their edge points [14]. A smaller mHD value suggests greater similarity between the compared volumes [7]. Based on mean mHD values in our study, similarity between GTV-CT/GTV-MRI and GTV-CT/GTV-PET_vis_ is suboptimal. It is probably because mHD is more responsive to shape changes of measured contours.

In our study we took into account only lymph nodes which were suspicious for malignancy on the basis of all three imaging methods. The purpose was to prevent false-positive results mainly obtained from PET. ^18^FDG-PET may over-stage patients by identifying benign inflammatory nodes as suspicious [30]. In case of metastatic lymph nodes, CT and MRI have a comparable sensitivity and specificity of 50–80% and 70–90%, respectively [31]. MRI, however, is the standard imaging method for the head and neck region because of excellent resolution of soft tissue and anatomical structures [9]. In the present study, nGTV-MRI were larger in 70% of cases compared to nGTV-CT. It can be related with higher soft tissue contrast in MRI vs. CT, as described above.

There are only few papers in which nGTV-PET obtained with manual delineation method were compared with nGTV-CT. In our study, differences between nGTV-PET_vis_ and nGTV-CT were statistically significant. nGTV-PET_vis_ were smaller that nGTV-CT in 80% of cases. It is related with low spatial resolution of PET, which resulted in the ability to detect lesions with dimensions above 5 mm. Moreover, PET visualizes foci of increased ^18^FDG uptake, not the entire lymphatic tissue. Bruela et al. [2] reported that nGTV-PET_vis_ were larger than nGTV-CT in 52% of cases. The authors explain this by differences in acquisition procedures and registration methods. Delouya et al. [15] found no significant difference between the nGTV delineated on CT and PET.

Among volumes of nGTV obtained from the fixed threshold method, the most significantly related with nGTV-CT were nGTV-PET_30%_ and nGTV-PET_40%_. Other authors demonstrated similar results; the threshold value chosen by them was about 36.6 ± 7.3% [9]. Ferrnando et al. [16] reported that volumes obtained from PET were smaller than nGTV-CT, when thresholds values were within the range of 20–50%. In our study 52.5% of all threshold measures are smaller than reference nGTV-CT.

Registration accuracy between GTV-CT and GTV-PET/MRI was rather low (mean DSC 0.55 and mHD 16.2 mm). Almost certainly it is related to the lack of patient immobilization during imaging studies and the time interval between CT and PET/MRI, which could influence observed changes in patient positioning.

In summary, tumor and lymph node volumes obtained from MRI were larger than reference volumes from CT, which is a result of better soft tissue imaging of MRI. Most of the^18^FDG-PET-based GTV were smaller in comparison to GTV-CT. ^18^FDG-PET significantly support target volume delineation and the combination with MRI decreases the risk of marginal miss and provides higher accuracy than other methods. The usefulness of PET/MRI increases in the event that a tumor or a lymph node is difficult to visualize using standard studies, which can be helpful in clinical practice. However, it is difficult to identify one of the contouring methods as the ideal method. Visual delineation is dependent on the observer. The threshold method is controversial, because volumes vary significantly, depending on the chosen threshold value. In this study we cannot point out one universal threshold value for the primary tumor of the tongue or for lymph nodes.

Further studies should include larger numbers of patient. PET/MRI should be made in the therapeutic position with immobilization of the patient head (individualized or fit mask), ideally, on the same or next day after simulation CT. Histopathological verification of the extent of tumor infiltration and metastatic lymph nodes observed in PET/MRI would be valuable in prospective trials. It would allow for the determination of the true biological GTV and verify PET/MRI accuracy in target volume imaging in cases of SCC of the tongue.

## Conclusion

Delineating GTV using ^18^FDG-PET/MRI requires a clearly established methodology. Combination of PET/MRI provides more information than other standard imaging studies, which might increase accuracy in target volume delineation. The most frequently matching threshold value was 30% for primary tumor delineation and for nGTV determination—30% and 40% of SUV_max_. Further prospective studies on a larger group of patients are needed to determine the best GTV delineation technique using innovative hybrid PET/MRI.

## Caption Electronic Supplementary Material

Tables series from statistical analyses of obtained data.
