# Supplementary material for: PET/MRI-guided GTV delineation during radiotherapy planning in patients with squamous cell carcinoma of the tongue
Source: Strahlenther Onkol. 2019 Jun 18;195(9):780–91. doi: 10.1007/s00066-019-01480-3 (PMC6704108; doi:10.1007/s00066-019-01480-3)
Supplement: Supplementary file 1 — Tables series from statistical analyses of obtained data. [file 66_2019_1480_MOESM1_ESM.docx]

|  | Primary tumor’s GTV size (cm^3)^ | | | | |
| --- | --- | --- | --- | --- | --- |
|  | Minimum | Average | Median | Maximum | ±SD |
| GTV CT | 4,1 | 21,2 | 15,8 | 82,2 | 23,67 |
| GTV MR | 4,5 | 25,3 | 12,3 | 93,6 | 29,27 |
| GTV PET vis | 1,7 | 23,2 | 12,1 | 100,6 | 31,94 |
| GTV PET 20% | 7,2 | 34,3 | 20,1 | 103,5 | 35,08 |
| GTV PET 30% | 2,6 | 19,1 | 9,3 | 66,9 | 23,77 |
| GTV PET 40% | 1,5 | 13,1 | 5,1 | 48,5 | 18,41 |
| GTV PET 50% | 0,95 | 9,5 | 2,8 | 36,7 | 14,13 |

SD – standard deviation, PET _vis_ – visual method; PET_20%_, PET_30%_, PET_40%_, PET_50%_ - volumes covered by 20%, 30%, 40%, 50% threshold of SUV_max_, respectively

Table 5. Statistical analysis of lymph nodes volumes (gross tumor volume, GTV) obtained from computed tomography (CT), magnetic resonance (MR) and 18-fluor-labeled fluorodeoxyglucose positron emitted tomography (18F-FDG-PET) taking into account the contouring method.

|  | Lymph nodes GTV size (cm^3)^ | | | | |
| --- | --- | --- | --- | --- | --- |
|  | Minimum | Average | Median | Maximum | ±SD |
| GTV CT | 1,1 | 3,4 | 3,3 | 5,8 | 1,82 |
| GTV MR | 2,1 | 4,3 | 3,2 | 9,1 | 2,62 |
| GTV PET vis | 0,4 | 1,9 | 1,1 | 5,1 | 1,75 |
| GTV PET 20% | 2,6 | 7,5 | 4,8 | 23,1 | 6,16 |
| GTV PET 30% | 1,5 | 4,4 | 2,8 | 9,3 | 3,08 |
| GTV PET 40% | 0,7 | 2,7 | 1,7 | 6,2 | 2,04 |
| GTV PET 50% | 0,4 | 1,5 | 1,0 | 3,6 | 1,18 |

SD – standard deviation, PET _vis_ – visual method; PET_20%_, PET_30%_, PET_40%_, PET_50%_ - volumes covered by 20%, 30%, 40%, 50% threshold of SUV_max_, respectively
